# Effects of direct tracheostomy on ventilator weaning in patients intubated for Guillain-Barré syndrome

**DOI:** 10.1016/j.aicoj.2026.100124

**Published:** 2026-07-28

**Authors:** Arnaud W. Thille, Constance Bayon, Marie Gauvrit, Clément Saccheri, Marie Labruyere, Gaetan Beduneau, Guilhem Wattecamps, Matthieu Henry, Eddine Bendiab, Benjamin Swinyard, Arnaud Gacouin, Adam Celier, Benjamin Foucou, Suzanne Champion, Anne-Fleur Haudebourg, Laurence Dangers, Olivier Plateker, Cedric Daubin, Lucie Fromont, Charlotte Guesdon, Emmanuel Vivier, Lev Volkov, Cyril Potier, Jeremy Bourenne, Charlotte Chiquet, Valentin Pointurier, Louis Chauvelot, Pierre Bailly, David Schnell, Nicholas Sedillot, Florian Reizine, Laurent Argaud, Faustine Reynaud, Juliette Pelle, Olivier Lesieur, Adrien Auvet, Guillaume Lacave, Marion Kernevez, Damien Contou, Guillaume Grillet, Arnaud Delahaye, Julio Badie, Jerome Devaquet, Hermine Chauvin, Alexandra Beurton, Emanuele Turbil, Jean-Philippe Rigaud, Stéphanie Ragot, Louis Chamblet, Sylvain Le Pape, Rémi Coudroy

**Affiliations:** a CHU de Poitiers, Service de Médecine Intensive Réanimation, Poitiers, France; b NSERM CIC 14-02, IS-ALIVE Research Group, University of Poitiers, France; c CHU de Lille, Service de Médecine Intensive Réanimation, Lille, France; d CHU de Nantes, Service de Médecine Intensive Réanimation, Nantes, France; e CHU de Nice, Service de Médecine Intensive Réanimation, Nice, France; f CHU de Dijon, Service de Médecine Intensive Réanimation, Dijon, France; g Univ Rouen Normandie, GRHVN UR 3830, CHU Rouen, Service de Médecine Intensive Réanimation, F-76000 Rouen, France; h CHU de Grenoble, Service de Médecine Intensive Réanimation, Grenoble, France; i CH de La Roche-sur-Yon, Service de Réanimation, La Roche-sur-Yon, France; j CHU de Montpellier, Service de Médecine Intensive Réanimation, Montpellier, France; k CHU d’Amiens, Service de Médecine Intensive Réanimation, Amiens, France; l CHU de Rennes, Service de Médecine Intensive Réanimation, Rennes, France; m AP-HP, Groupe Hospitalier Universitaire APHP-Sorbonne Université, Site Pitié-Salpêtrière, Service de Médecine Intensive et Réanimation (Département R3S), F-75013 Paris, France; n CH d’Avignon, Service de Réanimation, Avignon, France; o CHU de Bordeaux, Service de Médecine Intensive Réanimation, Bordeaux France; p AP-HP, Hôpital Henri Mondor, Service de Médecine Intensive Réanimation, Créteil, France; q CHU de la Réunion, Service de Médecine Intensive Réanimation, Saint Denis de la Réunion, France; r CHU de Limoges, Service de Médecine Intensive Réanimation, Limoges, France; s CHU de Caen Normandie, Service de Médecine Intensive Réanimation, Normandie Université, UNICAEN, Caen, France; t CHU d’Orléans, Service de Médecine Intensive Réanimation, Orléans, France; u CH de Pau, Service de Réanimation, Pau, France; v CH Saint Luc – Saint Joseph, Service de Réanimation, Lyon, France; w CH du Mans, Service de Médecine Intensive Réanimation, Le Mans, France; x AP-HP, Hôpital Bichat, Service de Médecine Intensive Réanimation, Paris, France; y AP-HM, Hôpital de la Timone, Service de Médecine Intensive Réanimation, Marseille, France; z CH de Lens, Service de Réanimation, Lens, France; A CH de Mulhouse, Service de Réanimation, Mulhouse, France; B HCL de Lyon, Hôpital de la Croix Rousse, Service de Médecine Intensive Réanimation, Lyon, France; C CHU de Brest, Service de Médecine Intensive Réanimation, Brest, France; D CH d’Angoulême, Service de Réanimation, Angoulême, France; E CHU de Bourg en Bresse, Service de Médecine Intensive Réanimation, Bourg en Bresse, France; F CH de Vannes, Service de Réanimation, Vannes, France; G HCL de Lyon, Hôpital Edouard Herriot, Service de Médecine Intensive Réanimation, Lyon, France; H CH de Saint Nazaire, Service de Réanimation, Saint Nazaire, France; I AP-HP, Hôpital Cochin, Service de Médecine Intensive Réanimation, Paris, France; J CH de La Rochelle, Service de Réanimation, La Rochelle, France; K CH de Dax, Service de Réanimation, Dax, France; L CH de Versailles, Service de Médecine Intensive Réanimation, Le Chesnay, France; M CH de Saintes, Service de Réanimation, Saintes, France; N CH d’Argenteuil, Service de Réanimation, Argenteuil, France; O CH de Lorient, Service de Réanimation, Lorient, France; P CH de Mont de Marsan, Service de Réanimation, Mont de Marsan, France; Q Hôpital Nord Franche-Comté, Service de Réanimation, Trévenans, France; R CH de Suresnes, Service de Réanimation, Suresnes, France; S AP-HM, Hôpital Nord, Service de Médecine Intensive Réanimation, Marseille, France; T AP-HP, Hôpital Tenon, Service de Médecine Intensive Réanimation, Paris, France; U CH d’Aurillac, Service de Réanimation, Aurillac, France; V CH de Dieppe, Service de Médecine Intensive Réanimation, Dieppe, France

**Keywords:** Guillain-Barré syndrome, Intensive Care Unit, Ventilator weaning, Tracheostomy, Mechanical ventilation

## Abstract

**Background:**

Whether or not a direct tracheostomy without any prior weaning attempt is beneficial in patients intubated for Guillain-Barré syndrome (GBS) remains to be determined. Our objective was to determine whether direct tracheostomy results in earlier or later ventilator weaning compared to standard weaning.

**Methods:**

Multicenter, retrospective cohort study including all patients intubated for GBS over a 10-year period (2014–2023). We compared patients having undergone direct tracheostomy without any prior weaning attempt with those having undergone standard weaning (spontaneous-breathing trial or extubation attempt). We also compared direct tracheostomy with tracheostomy after weaning failure. The primary outcome was the proportion of patients alive and free from mechanical ventilation 28 days after weaning initiation.

**Results:**

Overall, 221 patients (45%) underwent direct tracheostomy and 273 (55%) standard weaning. Compared to patients with standard weaning, those with direct tracheostomy were less likely to be alive and free from mechanical ventilation at day 28 (43% vs. 83%, p < 0.001). Using G-computation to estimate the marginal causal effect of the weaning strategy, standard weaning was more effective than direct tracheostomy in reducing the risk of remaining under mechanical ventilation at day 28: −39.5% [95% Confidence Interval, −47.3% to −31.5%]. Even when compared to patients having undergone tracheostomy after weaning failure, those with direct tracheostomy were less likely to be alive and free from mechanical ventilation 28 days after the tracheostomy procedure.

**Interpretation:**

A direct tracheostomy without any prior weaning attempt might significantly delay the ventilator weaning process in patients intubated for GBS.

**Clinical Trial Registration:**

NCT07022028

## Background

Severe forms of Guillain-Barré syndrome (GBS) may lead to acute respiratory failure and a need for intubation and mechanical ventilation [[1], [2], [3], [4]]. While respiratory failure is due primarily to respiratory muscle weakness, it can also be caused by bulbar weakness, leading to swallowing disorders that potentially result in aspiration pneumonia. Once intubated, these patients require particularly prolonged duration of mechanical ventilation in intensive care units (ICUs) and frequently present ventilator weaning difficulties [4]. The need for tracheostomy commonly appears while awaiting neuromuscular recovery sufficient to allow physiological spontaneous ventilation, as well as the restoration of effective cough and swallowing capacity [[4], [5], [6], [7], [8], [9], [10]]. However, data on ventilator weaning in these patients are scarce and primarily come from small-scale, often single-center, retrospective studies [[4], [5], [6], [7], [8], [9], [10]]. A recent narrative review on GBS acknowledged that due to the limited number of studies, ventilator weaning modalities were primarily based on expert opinions [4]. Moreover, a systematic review underscored a lack of studies showing the benefits of any specific weaning protocol in mechanically ventilated patients with neuromuscular disease [11].

In a recent, large-scale, multicenter, nationwide study, we described the characteristics of ventilator weaning in patients with GBS [12]. The main findings were that most patients experienced prolonged weaning, and that nearly 60% of patients underwent tracheostomy. Tracheostomy was performed either directly without any prior weaning attempt (i.e., neither spontaneous-breathing trial (SBT) nor extubation attempt), or after weaning failure (i.e., after repeated SBT failure or after extubation failure leading to reintubation). Whether or not a direct tracheostomy is beneficial in patients intubated for GBS remains to be determined. On the one hand, a direct tracheostomy in patients who could have been extubated through a standard weaning process may have detrimental effects by delaying ventilator weaning. On the other hand, a direct tracheostomy after intubation may result in early liberation from mechanical ventilation as compared to patients undergoing tracheostomy after weaning failure, which could actually be beneficial.

Therefore, we aimed to assess the effects of direct tracheostomy on outcomes of patients mechanically ventilated for GBS, as compared to patients having undergone a standard weaning process that included at least one SBT or an attempt at extubation. We also compared the effects of direct tracheostomy with tracheostomy performed after weaning failure.

## Methods

### Design and patients

Post-hoc analysis of a multicenter retrospective cohort study including all patients mechanically ventilated for at least 48 h due to GBS over a 10-year period (from January 2014 to December 2023) in 47 French ICUs [12]. Medical records were identified using the International Classification of Diseases 10th revision (G610). Patients who had been intubated for less than 48 h or for reasons other than neuromuscular disease were excluded.

### Ethics approval and consent to participate

The study was conducted in accordance with the Declaration of Helsinki. The study was approved by the independent institutional review board of Poitiers, and registered at http://www.clinicaltrials.gov (NCT07022028). In compliance with the national legislation regarding observational retrospective studies, the study was declared to European General Data Protection Regulation (MR004). An information letter was sent to all patients eligible in the study, and patients could be included on the condition that they did not object.

### Standard weaning process and tracheostomy

Each investigator was responsible for selecting all the data recorded in the case report form regarding ventilator weaning, especially that relating to the initial SBT, including the date, modality (T-piece or PSV), duration, and whether it was successful or not. We defined the standard weaning process as performing SBT using a T-piece or pressure-support ventilation, leading to the decision of extubation after a successful SBT. We also considered extubation without any prior SBT as a standard weaning process.

Tracheostomy could be performed according to the following two strategies: (1) direct tracheostomy without any prior weaning attempt (neither SBT nor extubation attempt), or (2) after standard weaning failure, i.e. either after repeated SBT failure (after the failure of at least two SBTs) without extubation attempt, or after extubation failure leading to reintubation.

### Outcomes

The primary outcome was the proportion of patients alive and free from mechanical ventilation 28 days after weaning initiation (defined as the initiation of a SBT, an extubation attempt, or tracheostomy), which is often used for tracheostomized patients who require prolonged duration of mechanical ventilation [13,14]. To mitigate immortal time bias — the risk that some patients may die before the event of interest occurs — we defined time point zero (T0) as the first weaning decision (i.e., the initiation of a SBT, an extubation attempt, or tracheostomy), rather than the time of intubation. This approach accounts for the possibility that some patients may die before weaning initiation. However, when comparing patients who underwent direct tracheostomy with those who underwent tracheostomy after weaning failure, we assessed the proportion of patients alive and free from mechanical ventilation 28 days after the tracheostomy procedure. The main secondary outcomes included total duration of mechanical ventilation, length of stay in ICU and mortality in ICU.

### Statistical analysis

All of the analyses were performed by the study statistician (L.C.). Continuous variables were expressed as mean ± standard deviation or median [interquartile range, IQR 25th-75th percentiles], and qualitative variables as number and percentage. Depending on data distribution, statistical comparisons of characteristics and outcomes were performed using Student's t-test or the Welch test or the Wilcoxon test for continuous variables, and using χ² tests or Fisher’s exact test for qualitative variables.

The primary outcome was compared between patients having undergone direct tracheostomy and those having undergone a standard weaning by means of the χ^2^ test. Curves assessing the probability of remaining under mechanical ventilation during the 28 days following weaning initiation were plotted and compared using Gray’s test, with death being treated as a competing risk.

The primary and secondary outcomes were also assessed between patients having undergone direct tracheostomy and those who having undergone tracheostomy after weaning failure, and between patients having undergone tracheostomy after SBT failure or after extubation failure.

To robustly estimate the marginal causal effect of the weaning strategy on the risk of remaining under mechanical ventilation at day 28 while addressing potential confounders, we used G-computation to estimate the average treatment effect, with missing data addressed through multiple imputation by chained equations (MICE; five imputed datasets). We first used least absolute shrinkage and selection operator (LASSO) regression to identify the most relevant covariates for inclusion in our model. An optimal LASSO penalty (λ) was estimated in the full population and then applied to select covariates within each imputed dataset. We subsequently fitted a logistic regression model, adjusted for direct tracheostomy assignment, clinically relevant confounders, and variables associated with the primary outcome, the objective being to predict the probability of remaining under mechanical ventilation 28 days after weaning initiation for each individual, both with and without direct tracheostomy scenarios. The average treatment effect was calculated as the mean difference between these predicted probabilities. Confidence intervals were estimated using bootstrap resampling (5 sets of 1,000 replications each). All statistical analyses were conducted using R software version 4.0.4 (R Foundation for Statistical Computing; https://www.R-project.org) and G-computation using GitHub-chupverse/computation: An R package for causal inference using G-computation. A two-tailed p value of less than 0.05 was considered statistically significant.

## Results

**Fig. 1:**
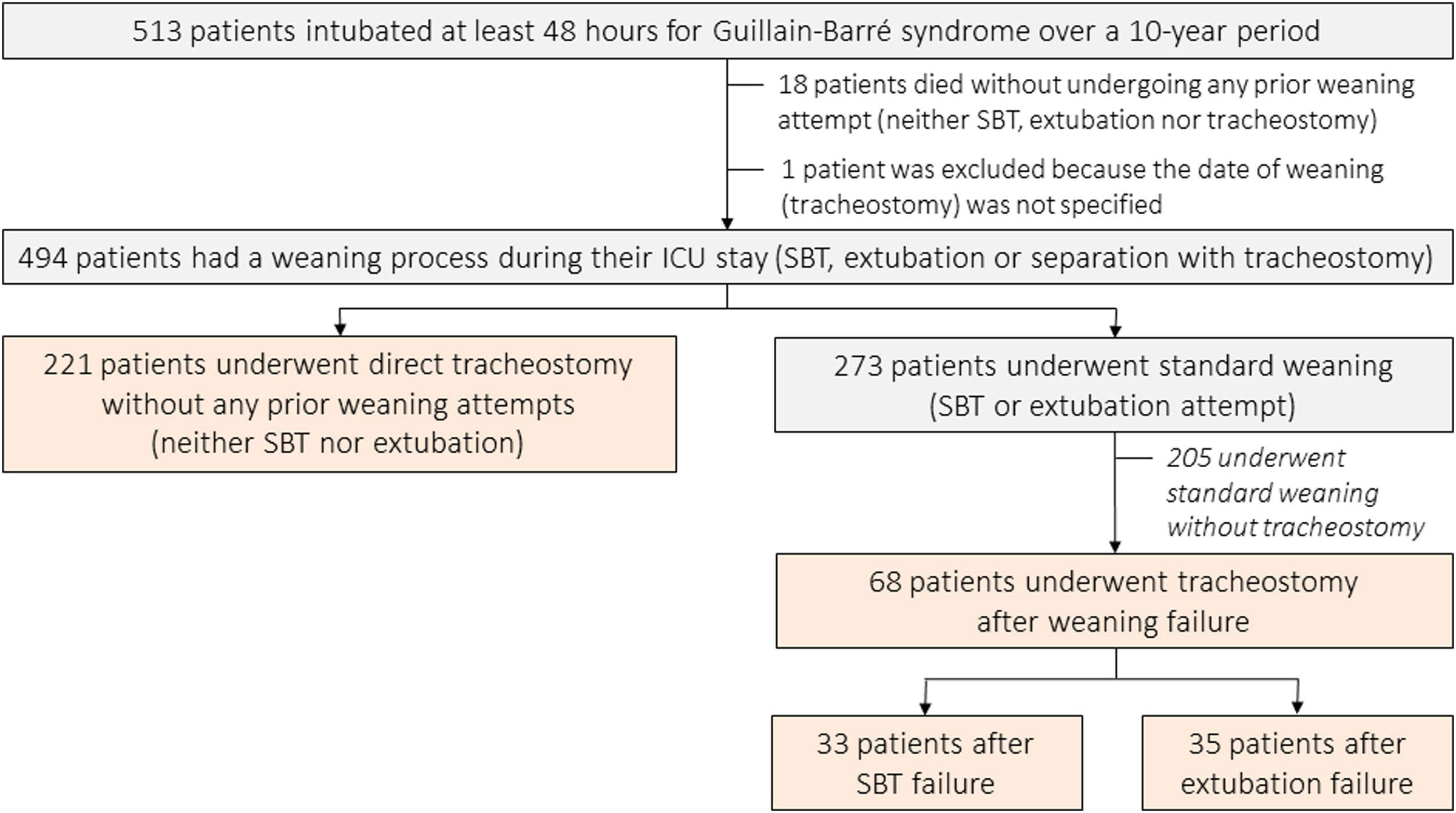
Patient flow chart. Of the 494 patients who had a weaning process during their ICU stay, a total of 289 patients (59%) underwent tracheostomy. Among them, 221 (77%) underwent direct tracheostomy without any prior weaning attempt and 68 (23%) underwent tracheostomy after weaning failure (i.e., after repeated spontaneous-breathing trial failure or extubation failure leading to reintubation).

Out of the 513 patients from 47 ICUs included in the original study, the 18 patients who died without having undergone any prior weaning attempt (neither SBT, extubation, nor liberation with tracheostomy) were excluded. Additionally, one patient who underwent direct tracheostomy was excluded due to missing data on the tracheostomy date. Therefore, 494 patients who underwent a weaning process during their ICU stay were included in the present analysis (Fig. 1). Among them, 221 patients (45%) underwent direct tracheostomy without any prior weaning attempt while 273 patients (55%) underwent standard weaning including SBT or extubation attempt. Among these patients, 68 underwent subsequent tracheostomy after weaning failure (23% of all tracheostomies), including 33 patients in whom tracheostomy was performed after repeated SBT failure and 35 patients after extubation failure leading to reintubation. All in all, 289 patients (59%) out of the 494 who had a weaning process underwent tracheostomy.

### Characteristics of patients with direct tracheostomy as compared to those with standard weaning

Compared to patients with standard weaning, those having undergone direct tracheostomy were more likely to be males and to have been diagnosed with GBS in the ICU (Table 1). They also more frequently received plasmatic exchange. Compared to patients with direct tracheostomy, those having undergone tracheostomy after weaning failure were more likely to have bulbar weakness (66% vs. 47%, p = 0.011) (Table 2). Compared to patients having undergone tracheostomy after extubation failure, those having undergone tracheostomy after repeated SBT failure had higher body-mass index and were more likely to have underlying chronic respiratory disease (Table 3).

**Table 1:** Comparison between patients who underwent direct tracheostomy versus all others who started a standard weaning process.

Patients with Guillain-Barré syndrome who started a weaning process (n = 494)	Direct tracheostomy (n = 221)	Standard weaning process (n = 273)	P value
Characteristics of the patients			
Age, years	56 ± 17	57 ± 19	0.843
Male sex, n (%)	144 (65%)	138 (51%)	**0.001**
Body-mass index, kg/m^2^	26 ± 5	26 ± 6	0. 748
SAPS II at admission, points	32 ± 15	33 ± 16	0.450
SOFA score at admission, points	2 [1–4]	2 [1–4]	0.621
Underlying chronic cardiac disease, n (%)	15 (7%)	29 (11%)	0.137
Underlying chronic respiratory disease, n (%)	15 (7%)	22 (8%)	0.594
Study period			0.507
2014−2018, n (%)	107 (48%)	124 (45%)	
2019−2023, n (%)	114 (52%)	149 (55%)	
Characteristics of the Guillain-Barré syndrome			
Diagnosis of GBS in ICU, n (%)	144/212 (68%)	148/267 (55%)	**0.005**
Bulbar weakness, n (%)	89/189 (47%)	127/252 (50%)	0.492
Antiganglioside antibodies, n (%)	58/214 (27%)	62/262 (24%)	0.390
Proteins in lumbar puncture, G/L	0.7 [0.4−1.1]	0.8 [0.5−1.5]	0.056
Axonal neuropathy on electromyogram, n (%)	118/211 (56%)	125/236 (53%)	0.531
Intravenous immunoglobulins, n (%)	213/219 (97%)	258/271 (95%)	0.241
Therapeutic plasma exchange, n (%)	52/220 (24%)	43/271 (16%)	**0.030**
Weaning Process			
At least one SBT performed, n (%)	0 (0%)	252 (92%)	**<0.001**
At least one extubation attempt, n (%)	0 (0%)	236 (86%)	**<0.001**
Tracheostomy performed, n (%)	221 (100%)	68 (25%)	**<0.001**
Outcomes			
Time from intubation to weaning initiation (T0), days	11 [5–18]	7 [4–13]	**<0.001**
Alive and free from mechanical ventilation 28 days after T0, n (%)	96 (43%)	228 (83%)	**<0.001**
Duration of mechanical ventilation after T0, days	32 [18−62]	4 [1–15]	**<0.001**
Total duration of mechanical ventilation, days	46 [29−77]	16 [9–27]	**<0.001**
ICU length of stay, days	57 [37−93]	22 [14−40]	**<0.001**
Death in ICU, n (%)	18 (8%)	12 (4%)	0.083

Values are given in mean ± standard deviation or median [interquartile range, 25–75 percentiles] and number (%). In bold are indicated p values considered as statistically significant (p < 0.05).

Abbreviations: SAPS II = Simplified Acute Physiology Score; SOFA = Sequential Organ Function Assessment; GBS = Guillain-Barré Syndrome; ICU = Intensive Care Unit, SBT = Spontaneous-Breathing Trial; T0 = weaning initiation (i.e., the initiation of a SBT, an extubation attempt or tracheostomy).

**Table 2:** Comparison between patients having undergone direct tracheostomy without any prior weaning attempt (neither spontaneous-breathing trial nor extubation attempt) versus those having undergone tracheostomy after weaning failure.

Patients who underwent tracheostomy in ICU (n = 289)	Direct tracheostomy (n = 221)	Tracheostomy after weaning failure (n = 68)	P value
Patient characteristics			
Age, years	56 ± 17	57 ± 19	0.640
Male sex, n (%)	144 (65%)	35 (51%)	**0.042**
Body-mass index, kg/m^2^	26 ± 5	26 ± 6	0.884
SAPS II at admission, points	32 ± 15	34 ± 17	0.356
SOFA score at admission, points	2 [1–4]	3 [1–4]	0.295
Underlying chronic cardiac disease, n (%)	15 (7%)	5 (7%)	0.791
Underlying chronic respiratory disease, n (%)	15 (7%)	5 (7%)	0.791
Characteristics of the Guillain-Barré syndrome			
Diagnosis of GBS in ICU, n (%)	144/212 (68%)	35/64 (55%)	0.052
Bulbar weakness, n (%)	89/189 (47%)	37/56 (66%)	**0.013**
Antiganglioside antibodies, n (%)	58/214 (27%)	11/64 (17%)	0.107
Proteins in lumbar puncture, G/L	0.7 (0.4−1.1)	0.9 (0.5−1.6)	0.144
Axonal neuropathy on electromyogram, n (%)	118/211 (56%)	37/62 (60%)	0.600
Intravenous immunoglobulins, n (%)	213//219 (97%)	63/66 (95%)	0.437
Therapeutic plasma exchange, n (%)	52/220 (24%)	17/66 (26%)	0.724
Weaning Process			
At least one SBT performed, n (%)	0 (0%)	64 (94%)	**<0.001**
At least one extubation attempt, n (%)	0 (0%)	35 (51%)	**<0.001**
Outcomes			
Time between intubation and tracheostomy, days	11 [5–18]	16 [10–26]	**<0.001**
Alive and free from MV 28 days after tracheostomy, n (%)	96 (43%)	40 (59%)	**0.026**
Duration of MV after tracheostomy, days	32 [18−62]	19 [8−46]	**0.002**
Liberation from MV before ICU discharge, n (%)	162 (73%)	58 (85%)	**0.043**
Decannulation before ICU discharge, n (%)	121 (55%)	40 (59%)	0.554
Total duration of mechanical ventilation (MV), days	46 [29−77]	38 [24−66]	0.183
ICU length of stay, days	57 [37−93]	52 [36−86]	0.484
Death in ICU, n (%)	18 (8.1%)	5 (7.4%)	0.833

Values are given in mean ± standard deviation or median [interquartile range, 25–75 percentiles] and number (%). In bold are indicated p values considered as statistically significant (p < 0.05).

Abbreviations: SAPS II = Simplified Acute Physiology Score; SOFA = Sequential Organ Function Assessment; GBS = Guillain-Barré Syndrome; ICU = Intensive Care Unit, SBT = Spontaneous-Breathing Trial; MV = Mechanical Ventilation.

**Table 3:** Comparison between patients with Guillain-Barré syndrome having undergone tracheostomy after spontaneous-breathing trial (SBT) failure versus those having undergone tracheostomy after extubation failure.

Patients having undergone tracheostomy after weaning failure (n = 68)	Tracheostomy after SBT failure (n = 33)	Tracheostomy after extubation failure (n = 35)	P value
Patient characteristics			
Age, years	57 ± 16	58 ± 21	0.789
Male sex, n (%)	18 (55%)	17 (49%)	0.622
Body-mass index, kg/m^2^	28 ± 8	24 ± 4	**0.025**
Obesity (BMI ≥ 30 kg/m^2^), n (%)	10/32 (31%)	2/34 (6%)	**0.008**
SAPS II at admission, points	31 ± 16	37 ± 18	0.133
SOFA score at admission, points	3 (1−3)	3 (1−5)	0.375
Underlying chronic cardiac disease, n (%)	2 (6%)	3 (9%)	1.000
Underlying chronic respiratory disease, n (%)	5 (15%)	0 (0%)	**0.023**
Characteristics of the Guillain-Barré syndrome			
Diagnosis of GBS in ICU, n (%)	16/32 (50%)	19/32 (59%)	0.451
Bulbar weakness, n (%)	19/29 (66%)	18/27 (67%)	0.928
Antiganglioside antibodies, n (%)	6/31 (19%)	5/33 (15%)	0.656
Proteins in lumbar puncture, G/L	0.8 (0.5−2.0)	0.9 (0.5−1.4)	0.941
Axonal neuropathy on electromyogram, n (%)	18/30 (60%)	19/32 (59%)	0.960
Intravenous immunoglobulins, n (%)	31/32 (97%)	32/34 (94%)	1.000
Therapeutic plasma exchange, n (%)	9/32 (28%)	8/34 (24%)	0.670
Weaning Process			
At least one SBT performed, n (%)	33 (100%)	31 (89%)	0.115
Outcomes			
Time between intubation and tracheostomy, days	15 [10–26]	16 [12–24]	0.902
Alive and free from MV 28 days after tracheostomy, n (%)	18 (55%)	22 (63%)	0.486
Duration of MV after tracheostomy, days	22 [7−57]	16 [9−38]	0.418
Liberation from MV before ICU discharge, n (%)	28 (85%)	30 (86%)	1.000
Decannulation before ICU discharge, n (%)	19 (58%)	21 (60%)	0.839
Total duration of mechanical ventilation (MV), days	45 [30−67]	34 [24−62]	0.484
ICU length of stay, days	58 [33−95]	49 [37−85]	0.859
Death in ICU, n (%)	2 (6%)	3 (9%)	1.000

Values are given in mean ± standard deviation or median [interquartile range, 25–75 percentiles] and number (%). In bold are indicated p values considered as statistically significant (p < 0.05).

Abbreviations: SAPS II = Simplified Acute Physiology Score; SOFA = Sequential Organ Function Assessment; GBS = Guillain-Barré Syndrome; ICU = Intensive Care Unit, SBT = Spontaneous-Breathing Trial; MV = Mechanical Ventilation.

### Primary and secondary outcomes

**Fig. 2:**
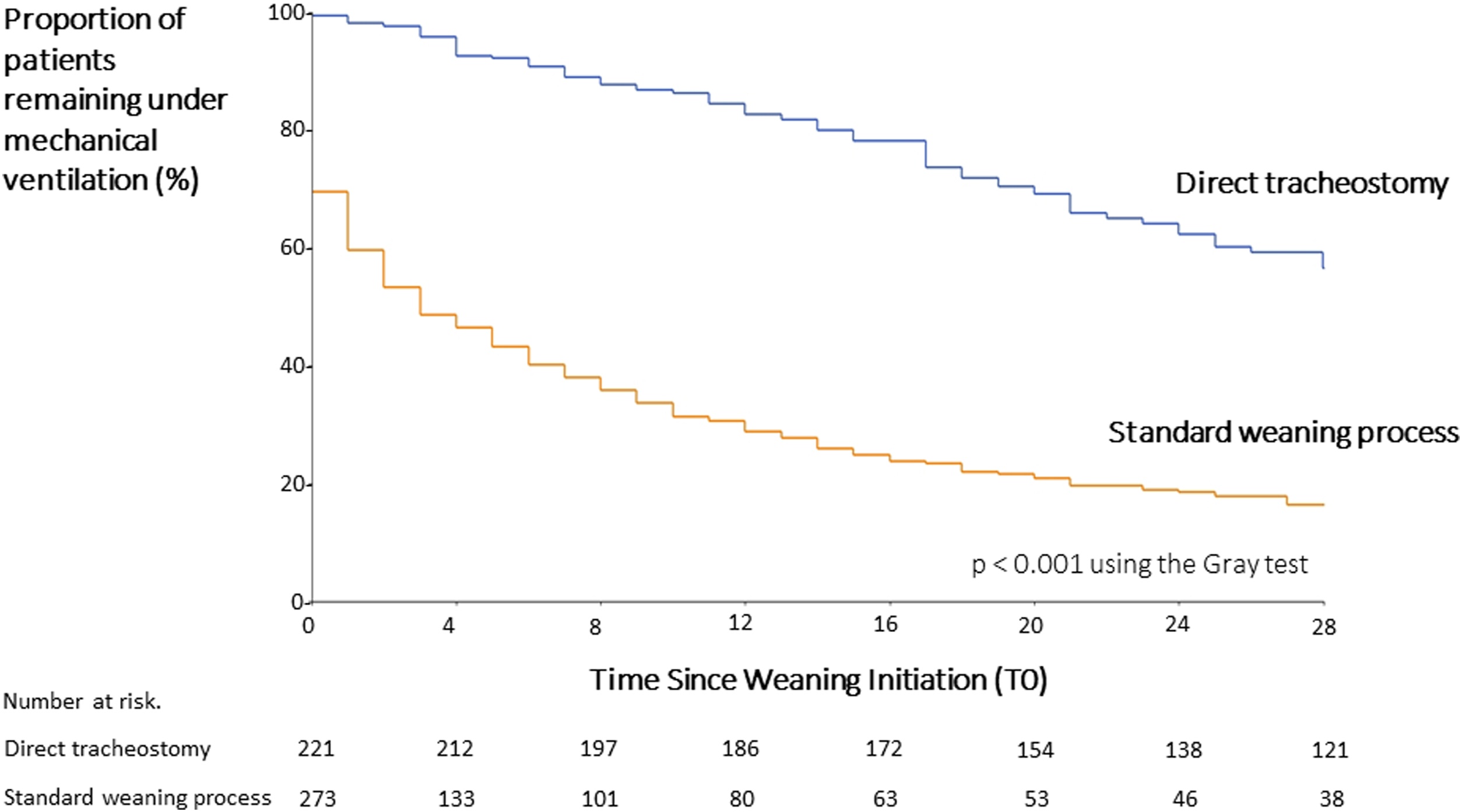
Curves showing the probability of remaining under mechanical ventilation within the 28 days following weaning initiation (T0) defined as the initiation of a spontaneous-breathing trial, an extubation attempt, or tracheostomy. Compared to patients having undergone standard weaning (orange curve), a lower proportion of patients having undergone direct tracheostomy (blue curve) were alive and free from mechanical ventilation within the 28 days following weaning initiation (43% vs. 83%, p < 0.001 using the Gray test).

Weaning initiation (T0) occurred earlier after intubation in the standard weaning group compared to the direct tracheostomy group (7 days in median [4–13] vs. 11 [5–18], p < 0.001) (Table 1). Compared to patients with standard weaning, patients having undergone direct tracheostomy were less likely to be alive and free from mechanical ventilation 28 days after weaning initiation (T0): 43% (96 out of 221 patients) vs. 83% (228 out of 273 patients), p < 0.001 using the χ² test and the Gray test (Table 1 and Fig. 2). Similarly, the proportion of patients alive and free from mechanical ventilation 28 days after intubation was lower with direct tracheostomy than with standard weaning: 22% (49 out of 221 patients) vs. 74% (201 out of 273 patients), p < 0.001 using the χ^2^ test. Using G-computation to estimate the marginal causal effect of the weaning strategy, standard weaning was more effective than direct tracheostomy in reducing the risk of remaining under mechanical ventilation 28 days after weaning initiation (T0), with a difference of −39.5% [95% CI, −47.3 to −31.5]. The logistic regression model using G-computation was adjusted on the following confounding variables (with the number of imputed missing data for each variable listed in parentheses): age, sex, body − mass index (n = 9), underlying chronic cardiac disease, severity SOFA score (n = 8), time from intubation to weaning initiation, diagnosis of GBS in ICU (n = 15) and axonal neuropathy on electromyogram (n = 47). The proportion of patients alive and free from mechanical ventilation 28 days after weaning initiation (T0) did not differ significantly between the first and second study periods: 63% (145 out of 231 patients) from 2014 to 2018 versus 68% (179 out of 263 patients) from 2019 to 2023 (p = 0.217). Patients having undergone direct tracheostomy had also longer durations of mechanical ventilation and ICU length of stay than those with standard weaning (Table 1). In-ICU mortality tended to be lower with standard weaning than with direct tracheostomy, albeit non-significantly.

**Fig. 3:**
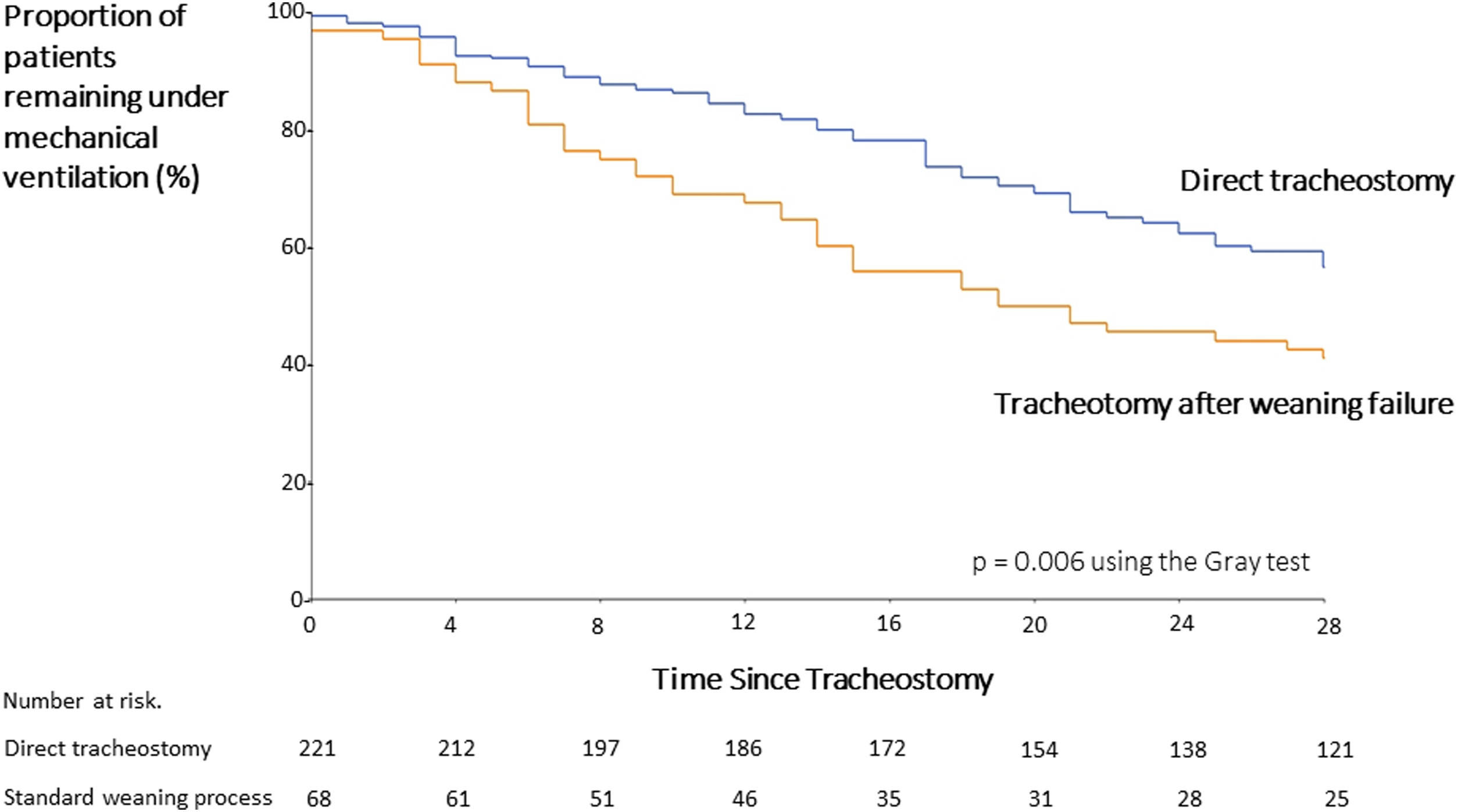
Curves showing the probability of remaining under mechanical ventilation within the 28 days following the tracheostomy procedure. Compared to patients having undergone tracheostomy after weaning failure (orange curve), i.e., after repeated spontaneous-breathing trial failure or extubation failure, a lower proportion of patients having undergone direct tracheostomy (blue curve) were alive and free from mechanical ventilation within the 28 days following the tracheostomy procedure (43% vs. 59%, p = 0.006 using the Gray test).

Although direct tracheostomy was performed earlier than tracheostomy after weaning failure (11 days in median [5–18] vs. 16 [10–26], p < 0.001), the proportion of patients who were alive and free from mechanical ventilation 28 days after the tracheostomy procedure was significantly lower with direct tracheostomy than with tracheostomy after weaning failure: 43% (96 out of 221 patients) vs. 59% (40 out of 68 patients), p = 0.026 using the χ² test p = 0.006 using the Gray test **(**Table 2 and Fig. 3**)**. Moreover, a lower proportion of patients with direct tracheostomy were completely liberated from mechanical ventilation at ICU discharge (73% vs. 85%, p = 0.043) **(**Table 2**)**. No difference was found in outcomes between patients having undergone tracheostomy after SBT failure or after extubation failure (Table 3).

### Center effect

As shown in the supplemental data (Figure S1), standard weaning was predominant in some centers, while direct tracheostomy was routinely performed in others, suggesting that the decision to perform a direct tracheostomy was not solely based on disease severity. Whereas standard weaning was often associated with a higher proportion of patients who were alive and free from mechanical ventilation 28 days after weaning initiation, direct tracheostomy was not associated with better outcomes in any center (Figure S2).

## Discussion

In this multicenter retrospective study including 494 patients with GBS who started a weaning process during their ICU stay, nearly half of them (45%) underwent direct tracheostomy without any prior weaning attempt. The main finding of the study is that patients having undergone direct tracheostomy were less likely to be liberated from mechanical ventilation 28 days after weaning initiation than those having undergone a standard weaning process including at least one SBT or an extubation attempt. These patients also had a longer duration of mechanical ventilation and a longer ICU length of stay than those having undergone standard weaning. Even when compared to patients having undergone tracheostomy after weaning failure, direct tracheostomy was less effective in reducing the risk of remaining on mechanical ventilation 28 days after the tracheostomy procedure. Patients having undergone tracheostomy after weaning failure were more likely to be completely liberated from the ventilator at ICU discharge than those with direct tracheostomy. These findings reinforce the idea that standard weaning is more effective than direct tracheostomy, even when weaning difficulties persist, and that standard weaning leads to a subsequent tracheostomy.

### Tracheostomy in patients with GBS

In our study, 289 (59%) of the 494 patients who had a weaning process eventually underwent tracheostomy (direct or after weaning failure). Even though this rate seems high, it is lower than those reported in the literature, which range from 57% (very similar to our study) to more than 90% [[5], [6], [7], [8], [9]]. Although a tracheostomy can improve patient comfort, contribute to work of breathing, promote out-of-bed positioning [[15], [16], [17]], and facilitate oral hygiene, oral feeding, and communication [18], while enabling better assessment of swallowing and bulbar function, the ideal timing remains debated [13,14,[19], [20], [21]]. Theoretically, a direct tracheostomy after intubation may hasten the ventilator weaning process. However, a direct tracheostomy without prior weaning attempt may result in unnecessary tracheostomy in patients who could have been extubated without major weaning difficulties. Mechanical ventilation can be particularly prolonged in patients with GBS ranging from a few days to several months and the vast majority of these patients are eventually tracheostomized [4,7,8,22]. Although several studies have shown that severe limb weakness was a strong predictor of prolonged mechanical ventilation [7,22], it is difficult to accurately predict early on which patients will experience major weaning difficulties and prolonged duration of mechanical ventilation. Conversely, it is easier to decide to perform a tracheotomy once weaning failure has been proven. The impact on outcomes in the general ICU patient population of early tracheostomy (i.e., within the first few days after intubation, without prior weaning attempt) versus late tracheostomy (usually after weaning failure) remains contradictory [13,14,[19], [20], [21]]. While some studies have shown beneficial effects of early tracheostomy on ventilator-associated pneumonia, ventilation duration and mortality [13,14], other studies have shown no difference [[19], [20], [21]]. In patients with myasthenia gravis, a retrospective multicenter study reported that patients with early tracheostomy (fewer than 10 days) had shorter duration of mechanical ventilation and ICU length of stay than those with late tracheostomy [23]. However, even though a given tracheostomy was performed early, it is unclear whether it was carried out directly, without any prior weaning attempt, or after weaning failure. Furthermore, and consistent with our results, this study showed that patients having undergone standard weaning had a shorter duration of mechanical ventilation than those having undergone tracheostomy [23]. As regards patients with GBS, only one study specified that tracheostomy was directly performed without prior weaning attempt in 80% of cases [6], which is very close to our rate of 77%. Moreover, the median time from intubation to tracheostomy was 11 days when performed directly without weaning attempt and 16 days when performed after weaning failure, which is close to the time frame (9–14 days) reported in previous studies [5,7,24].

### Strengths of the study

One of the strengths of this study is that it is the largest to date reporting on weaning modalities and tracheostomy in patients with GBS across numerous ICUs, including both teaching and non-teaching hospitals, thereby enhancing its generalizability. We focused on clinically relevant outcomes, particularly complete liberation from mechanical ventilation at day 28, which is a patient-centered outcome frequently used to assess weaning in tracheostomized patients [13,14]. Lastly, we compared direct tracheostomy not only with standard weaning but also with tracheostomy after weaning failure, thereby underlining the impact of timing and approach. In fact, comparing patients having undergone tracheostomy after weaning failure helps identify the most severe subgroup in the standard weaning population.

### Limitations of the study

The main limitation is obviously the retrospective nature of our study, with inherent information bias, and we cannot rule out remaining confounding factors, even after adjustment. In fact, patients selected for direct tracheostomy may have had more severe disease, particularly involving more severe muscle weakness, ineffective cough, or more frequent swallowing disorders. A major limitation is the lack of data on respiratory function, including the intensity of respiratory muscle weakness, the level of ventilatory support required, vital capacity, and cough strength, all of which could impact the decision for tracheostomy. Moreover, since we only collected weaning modalities, we cannot rule out the possibility that some patients underwent direct tracheostomy because they could not tolerate partial ventilation and had not undergone SBTs. However, in the first few days following intubation, it is difficult to predict how quickly the patient will recover [7]. Notwithstanding, we included a relatively homogeneous subset of patients with particularly severe cases, who were intubated in ICUs, and we collected instances of bulbar weakness, which may be a major determinant for tracheostomy [9]. Patients having undergone direct tracheostomy were less likely to have bulbar weakness than those having undergone tracheostomy after weaning failure, suggesting, on the contrary, a less severe disease. Furthermore, axonal neuropathy, which is a major factor in delayed muscle strength recovery and prolonged mechanical ventilation due to potential irreversible damage [4], was no more prevalent in patients with direct tracheostomy.

Despite that, variability in weaning protocols across ICUs, particularly the decision to perform a direct tracheostomy, may have been influenced by many factors including clinician judgment, local practices, or the availability of weaning facilities, and could differ widely from center to center. The lack of a standardized approach to SBT initiation or direct tracheostomy is undoubtedly a significant source of bias. The timing of the direct tracheostomy was also later than the initiation of standard weaning, which could introduce a significant bias against direct tracheostomy. However, the proportion of patients alive and free from mechanical ventilation was significantly higher with standard weaning than with direct tracheostomy, even when comparing the time frame 28 days after intubation rather than after weaning initiation. We also observed a higher incidence of direct tracheostomy in males. We cannot determine whether this is related to males exhibiting more severe forms of GBS or if management differs according to sex due to cognitive bias, as has been reported regarding cardiac diseases, for which management could be less aggressive and effective in females [[25], [26], [27]]. To mitigate the impact of these confounding factors on causal inference, we applied robust statistical methods using G-computation to estimate the causal effect of different weaning strategies, which is likely the most appropriate way to control confounding bias [28]. However, the lack of randomization did not allow for complete elimination of all potential confounding factors, and unmeasured variables could still obviously have influenced the choice of weaning strategy.

Another major limitation is the lack of follow-up beyond ICU discharge, and especially until complete decannulation in weaning centers. The length of stay in the ICU did not differ between patients who underwent direct tracheostomy and those who underwent tracheostomy after weaning failure. However, ICU discharge of patients who are still on mechanical ventilation or who are still tracheostomized may vary across centers. In fact, liberation from mechanical ventilation occurred earlier in cases of tracheostomy after weaning failure than in cases of direct tracheostomy, which seems more relevant. Although around three quarters of patients (220 out of 290 patients, 76%) were completely liberated from the ventilator at time of ICU discharge, we did not assess long-term functional recovery, particularly the resumption of walking, quality of life, post-traumatic stress disorders, or late complications of tracheostomy, especially tracheal stenosis.

### Clinical implications and future research

Our findings strongly support a standard weaning process including SBTs followed by extubation if successful, and limiting tracheostomy to patients with repeated SBT failure or extubation failure leading to reintubation. In fact, standard weaning appears more effective than direct tracheostomy in achieving earlier ventilator weaning in patients with GBS. By contrast, direct tracheostomy may delay liberation from mechanical ventilation and prolong ICU stay. In accordance with experts in a recent review [4], our findings suggest that tracheostomy should rather be considered for patients who have failed multiple SBTs or have experienced at least one episode of extubation failure. To confirm these findings and establish optimal weaning protocols for patients with GBS, a clinical trial comparing standard weaning versus direct tracheostomy in this specific population would be warranted. In addition to the primary outcome of complete liberation from mechanical ventilation, long-term outcomes should be included until there is complete recovery of spontaneous breathing without tracheostomy, as well as quality of life.

## Conclusions

A direct tracheostomy without any prior weaning attempt might significantly delay the ventilator weaning process in patients intubated for Guillain-Barré syndrome.

## Authors' contributions

AWT and LC had full access to all of the data in the study. AWT take responsibility for the integrity of the data and the accuracy of the data analysis. AWT designed the study. AWT wrote the manuscript. LC and SR performed statistical analysis. All authors collected data of patients in their center, contributed to drafting of the work, revising it critically for important intellectual content and approved the final version of the manuscript. All authors give their agreement to be accountable for all aspects of the work, and ensure the accuracy and integrity of any part of the work.

## Consent for publication

Not applicable.

## Ethics approval and consent to participate

The study was approved by the independent institutional review board of Poitiers, and registered at http://www.clinicaltrials.gov (NCT07022028). In compliance with the national legislation regarding observational retrospective studies, the study was declared to European General Data Protection Regulation (MR004). An information letter was sent to all patients eligible in the study, and patients could be included on the condition that they did not object.

## Funding

CHU de Poitiers.

## Availability of data materials

The datasets used and analyzed during the current study are available from the corresponding author on reasonable request.

Clinical Trial Registration: NCT07022028.

## Data availability

The data that has been used is confidential.

Data will be made available on request.

## Declaration of competing interest

All authors declare that they have no conflicts of interest related to the manuscript.
