# Supplementary material for: Effects of direct tracheostomy on ventilator weaning in patients intubated for Guillain-Barré syndrome
Source: Ann Intensive Care. 2026 Jul 28;16:100124. doi: 10.1016/j.aicoj.2026.100124 (PMC13463968; doi:10.1016/j.aicoj.2026.100124)

Effects of direct tracheostomy on ventilator weaning in patients intubated for Guillain-Barré syndrome

Authors:

Arnaud W. THILLE[1,2]; Constance BAYON[3]; Marie GAUVRIT[4]; Clément SACCHERI[5]; Marie LABRUYERE[6]; Gaetan BEDUNEAU[7]; Guilhem WATTECAMPS[8]; Matthieu HENRY[9]; Eddine BENDIAB[10]; Benjamin SWINYARD[11]; Arnaud GACOUIN[12]; Adam CELIER[13]; Benjamin FOUCOU[14]; Suzanne CHAMPION[15]; Anne-Fleur HAUDEBOURG[16]; Laurence DANGERS[17]; Olivier PLATEKER[18]; Cedric DAUBIN[19]; Lucie FROMONT[20]; Charlotte GUESDON[21]; Emmanuel VIVIER[22]; Lev VOLKOV[23]; Cyril POTIER[24]; Jeremy BOURENNE[25]; Charlotte CHIQUET[26]; Valentin POINTURIER[27]; Louis CHAUVELOT[28]; Pierre BAILLY[29]; David SCHNELL[30]; Nicholas SEDILLOT[31]; Florian REIZINE[32]; Laurent ARGAUD[33]; Faustine REYNAUD[34]; Juliette PELLE[35]; Olivier LESIEUR[36]; Adrien AUVET[37]; Guillaume LACAVE[38]; Marion KERNEVEZ[39]; Damien CONTOU[40]; Guillaume GRILLET[41]; Arnaud DELAHAYE[42]; Julio BADIE[43]; Jerome DEVAQUET[44]; Hermine CHAUVIN[45]; Alexandra BEURTON[46]; Emanuele TURBIL[47]; Jean-Philippe RIGAUD[48]; Stéphanie RAGOT[2], Louis CHAMBLET[2]; Sylvain LE PAPE[1,2]; and Rémi COUDROY[1,2];

SUPPLEMENTARY MATERIAL

Table S1: Comparison between patients who underwent direct tracheostomy versus all others who started a standard weaning process among the 243 patients who had axonal neuropathy.

Table S2: Comparison between patients who underwent direct tracheostomy versus all others who started a standard weaning process among the 216 patients who had bulbar weakness.

Figure S1: Caterpillar plot showing the difference in the weaning process across centers. While standard weaning was predominant in some centers (bottom left), direct tracheostomy was predominant in others (top right).

Figure S2: Caterpillar plot showing the difference in the proportion of liberation from mechanical ventilation across centers. Whereas standard weaning was often associated with a higher proportion of patients alive and free from mechanical ventilation 28 days after initiation of weaning (bottom left). Direct tracheostomy was never associated with better outcomes (top right).

Table S1: Comparison between patients who underwent direct tracheostomy versus all others who started a standard weaning process among the 243 patients who had axonal neuropathy.

**Patients with Guillain-Barré syndrome and axonal neuropathy who started a weaning process (n=243)**	**Direct tracheostomy (n=118)**	**Standard weaning process (n=125)**	**P value**
**Characteristics of the patients**			
Age, years	58 ± 16	57 ± 19	0.815
Male sex, n (%)	78 (66%)	62 (50%)	**0.009**
BMI	26 ± 6	26 ± 7	0.526
SAPS II at admission, points	34 ± 16	34 ± 16	0.881
SOFA score at admission, points	3 [1-4]	3 [1-4]	0.904
Underlying chronic cardiac disease,	7 (6%)	15 (12%)	0.099
Underlying chronic respiratory disease, n (%)	6 (5%)	11 (9%)	0.256
**Characteristics of the Guillain Barré syndrome**			
Diagnosis of GBS in ICU, n (%)	83/111 (75%)	73/121 (60%)	**0.019**
Bulbar weakness, n (%)	48/103 (46.6%)	65/118 (55%)	0.208
Antiganglioside antibodies, n (%)	31/113 (27.4%)	41/123 (33%)	0.325
Proteins in lumbar puncture, G/L	0.6 [0.4-1.1]	0.8 [0.4-1.4]	0.237
Intravenous immunoglobulins, n (%)	116 (98%)	120 (96%)	0.448
Therapeutic plasma exchange, n (%)	29 (25%)	22 (18%)	0.182
**Weaning Process**			
At least one SBT performed, n (%)	0 (0%)	113 (90%)	**<0.001**
At least one extubation attempt, n (%)	0 (0%)	104 (83%)	**<0.001**
Tracheostomy performed, n (%)	118 (100%)	37 (30%)	**<0.001**
**Outcomes**			
Time from intubation to weaning initiation (T0), days	11 [6-19]	8 [4-13]	**0.014**
Alive and free from mechanical ventilation 28 days after T0, n (%)	44 (37%)	97 (78%)	**<0.001**
Duration of mechanical ventilation after T0, days	34 [18-69]	6 [1-19]	**<0.001**
Total duration of mechanical ventilation, days	50 [30-87]	17 [11-32]	**<0.001**
ICU length of stay, days	63 [38-109]	23 [15-43]	**<0.001**
Death in ICU, n (%)	13 (11%)	10 (8%)	0.422

Values are given in mean ± standard deviation or median [interquartile range, 25-75 percentiles] and number (%). In bold are indicated p values considered as statistically significant (p < 0.05).
Abbreviations*: SAPS II = Simplified Acute Physiology Score; SOFA = Sequential Organ Function Assessment; GBS = Guillain-Barré Syndrome; ICU = Intensive Care Unit, SBT = Spontaneous-Breathing Trial; T0 = weaning initiation (i.e., the initiation of a SBT, an extubation attempt or tracheostomy).*

Table S2: Comparison between patients who underwent direct tracheostomy versus all others who started a standard weaning process among the 216 patients who had bulbar weakness.

**Patients with Guillain-Barré syndrome and bulbar weakness who started a weaning process (n=216)**	**Direct tracheostomy (n=89)**	**Standard weaning process (n=127)**	**P value**
**Characteristics of the patients**			
Age, years	54 ± 19	55 ± 19	0.707
Male sex, n (%)	54 (60.7%)	60 (47.2%)	0.052
BMI	25 ± 4	26 ± 6	0.169
SAPS II at admission, points	30 ± 14	31 ± 16	0.705
SOFA score at admission, points	2 (1-4)	2 (1-3)	0.168
Underlying chronic cardiac disease,	8 (9.0%)	12 (9.4%)	0.909
Underlying chronic respiratory disease, n (%)	3 (3.4%)	4 (3.1%)	1.000
**Characteristics of the Guillain Barré syndrome**			
Diagnosis of GBS in ICU, n (%)	50/84 (59.5%)	65/124 (52.4%)	0.312
Antiganglioside antibodies, n (%)	23/86 (26.7%)	34/125 (27.2%)	0.942
Proteins in lumbar puncture, G/L	0.6 [0.4-1.4]	0.7 [0.4-1.3]	0.583
Axonal neuropathy on electromyogram, n (%)	48/85 (56.5%)	65/109 (59.6%)	0.658
Intravenous immunoglobulins, n (%)	87 (97.8%)	119 (93.7%)	0.202
Therapeutic plasma exchange, n (%)	25 (28.1%)	33 (26.0%)	0.731
**Weaning Process**			
At least one SBT performed, n (%)	0 (0.0%)	122 (96.1%)	**<0.001**
At least one extubation attempt, n (%)	0 (0.0%)	106 (83.5%)	**<0.001**
Tracheostomy performed, n (%)	89 (100%)	37 (29.1%)	**<0.001**
**Outcomes**			
Time from intubation to weaning initiation (T0), days	12 (6-17)	9 (4-14)	0.056
Alive and free from mechanical ventilation 28 days after T0, n (%)	41 (46.1%)	100 (78.7%)	**<0.001**
Duration of mechanical ventilation after T0, days	31 (14-68)	7 (1-19)	**<0.001**
Total duration of mechanical ventilation, days	45 (24-83)	18 (12-36)	**<0.001**
ICU length of stay, days	58 (35-102)	27 (17-49)	**<0.001**
Death in ICU, n (%)	5 (5.6%)	8 (6.3%)	0.836

Values are given in mean ± standard deviation or median [interquartile range, 25-75 percentiles] and number (%). In bold are indicated p values considered as statistically significant (p < 0.05).
Abbreviations*: SAPS II = Simplified Acute Physiology Score; SOFA = Sequential Organ Function Assessment; GBS = Guillain-Barré Syndrome; ICU = Intensive Care Unit, SBT = Spontaneous-Breathing Trial; T0 = weaning initiation (i.e., the initiation of a SBT, an extubation attempt or tracheostomy).*

Figure S1: Caterpillar plot showing the difference in the weaning process across centers. While standard weaning was predominant in some centers (bottom left), direct tracheostomy was predominant in others (top right).

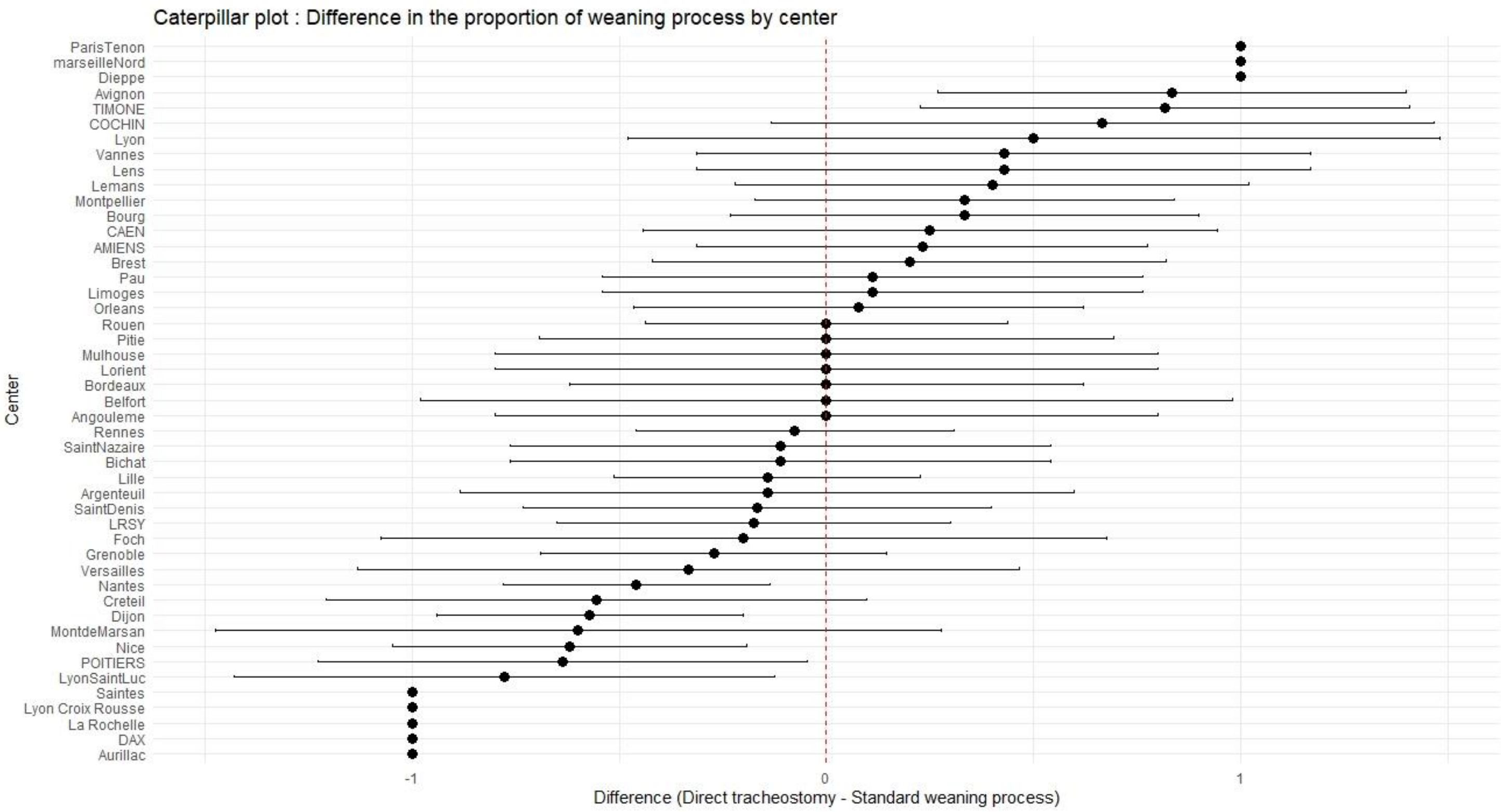

Figure S2: Caterpillar plot showing the difference in the proportion of liberation from mechanical ventilation across centers. Whereas standard weaning was often associated with a higher proportion of patients alive and free from mechanical ventilation 28 days after weaning initiation (bottom left). Direct tracheostomy was never associated with better outcomes (top right).

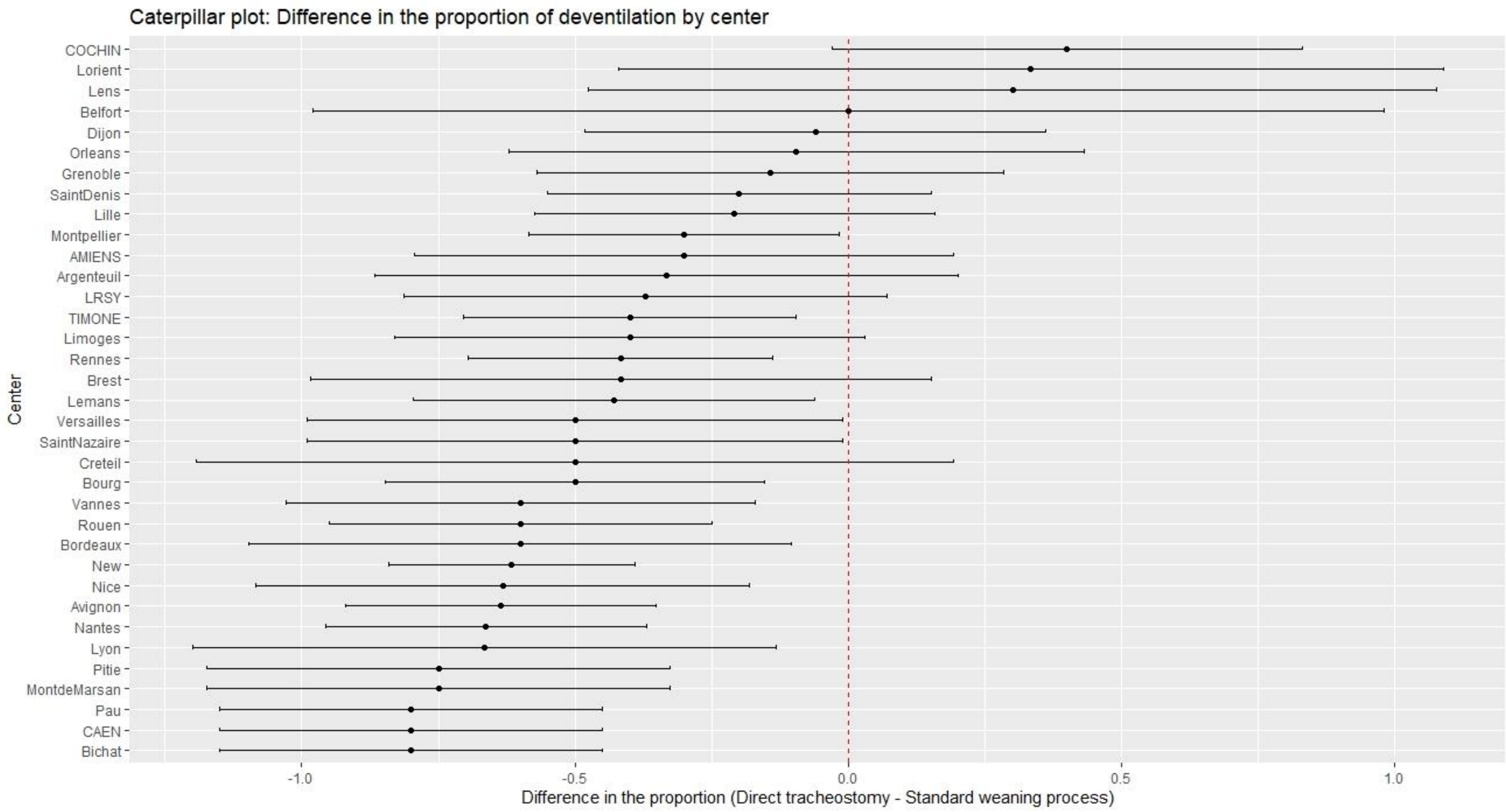

Supplement: Supplementary file 1 [file mmc1.pdf]
